# Mitochondrial function assessed by ^31^P MRS and BOLD MRI in non‐obese type 2 diabetic rats

**DOI:** 10.14814/phy2.12890

**Published:** 2016-08-10

**Authors:** Yuchi Liu, Xunbai Mei, Jielei Li, Nicola Lai, Xin Yu

**Affiliations:** ^1^ Department of Biomedical EngineeringCase Western Reserve UniversityClevelandOhio; ^2^ Case Center for Imaging ResearchCase Western Reserve UniversityClevelandOhio; ^3^ Department of Electrical and Computer Engineering and Biomedical Engineering InstituteOld Dominion UniversityNorfolkVirginia; ^4^ Department of RadiologyCase Western Reserve UniversityClevelandOhio; ^5^ Department of Physiology and BiophysicsCase Western Reserve UniversityClevelandOhio

**Keywords:** ^31^P magnetic resonance spectroscopy, blood oxygen level‐dependent MRI, ischemia reperfusion, mitochondrial oxidative capacity

## Abstract

The study aims to characterize age‐associated changes in skeletal muscle bioenergetics by evaluating the response to ischemia‐reperfusion in the skeletal muscle of the Goto‐Kakizaki (GK) rats, a rat model of non‐obese type 2 diabetes (T2D). ^31^P magnetic resonance spectroscopy (MRS) and blood oxygen level‐dependent (BOLD) MRI was performed on the hindlimb of young (12 weeks) and adult (20 weeks) GK and Wistar (control) rats. ^31^P‐MRS and BOLD‐MRI data were acquired continuously during an ischemia and reperfusion protocol to quantify changes in phosphate metabolites and muscle oxygenation. The time constant of phosphocreatine recovery, an index of mitochondrial oxidative capacity, was not statistically different between GK rats (60.8 ± 13.9 sec in young group, 83.7 ± 13.0 sec in adult group) and their age‐matched controls (62.4 ± 11.6 sec in young group, 77.5 ± 7.1 sec in adult group). During ischemia, baseline‐normalized BOLD‐MRI signal was significantly lower in GK rats than in their age‐matched controls. These results suggest that insulin resistance leads to alterations in tissue metabolism without impaired mitochondrial oxidative capacity in GK rats.

## Introduction

Type 2 diabetes (T2D) is a common form of diabetes mellitus. It is characterized by hyperglycemia as a consequence of insulin resistance and relative insulin deficiency (Hoda and Hoda 2005). Skeletal muscle is the major site of insulin‐mediated glucose uptake in the postprandial state (De Fronzo and Tripathy 2009). Emerging evidence suggests that impaired muscle metabolism plays a major role in the pathogenesis of T2D, and reducing mitochondrial oxidative damage may be a therapeutic target for preventing reduction in muscle mitochondrial function in T2D (Petersen et al. 2004). Metabolic dysfunction in skeletal muscle has been documented in the development of T2D (Kelley et al. 2002; Lowell and Shulman 2005; Ritov et al. 2005; Befroy et al. 2007; Perry et al. 2013). Previous studies have reported reduced mitochondria electron transport chain activity (Ritov et al. 2005), low mitochondrial content (Kelley et al. 2002; Boushel et al. 2007), and smaller skeletal muscle mitochondria (Ritov et al. 2005) in T2D patients. A defect in mitochondrial oxidative phosphorylation in both diabetic patients and animal models of T2D has also been reported (Befroy et al. 2007; Perry et al. 2013). However, conflicting results have also been reported that suggest normal mitochondrial function in T2D patients (Boushel et al. 2007; De Feyter et al. 2008a).

One key parameter used in evaluating mitochondrial function is the mitochondrial oxidative capacity. Magnetic resonance imaging and spectroscopy (MRI/MRS) can provide noninvasive and dynamic tools that are well suited to assessing mitochondrial function in vivo. Phosphorus‐31 (^31^P) MRS offers direct quantification of the high‐energy phosphate metabolites such as adenosine triphosphate (ATP) and phosphocreatine (PCr) (Perry et al. 2013). In particular, monitoring the depletion and resynthesis of PCr during exercise‐recovery or ischemia‐reperfusion by dynamic ^31^P MRS allows the assessment of mitochondrial oxidative capacity in the muscle (Meyer 1988; Paganini et al. 1997). Furthermore, blood oxygenation level‐dependent (BOLD) MRI allows the assessment of muscle oxygenation *in vivo* by detecting changes in the oxygenated versus deoxygenated hemoglobin ratio (Ogawa et al. 1990). The technique has been used to evaluate muscle oxygenation following arterial occlusion (Ledermann et al. 2006). Simultaneous assessment of mitochondrial function and tissue oxygenation may lead to a more comprehensive understanding of the energetics of skeletal muscle in T2D. In addition, these noninvasive approaches enable longitudinal studies aimed at delineating the time course of metabolic changes in T2D.

To date, most studies using animal models of T2D have focused on obese T2D rodents (King 2012). While insulin resistance is frequently associated with obesity, not all T2D patients are obese. Hence, a lean animal model of T2D is of value to studying mitochondrial function in non‐obese T2D. The Goto‐Kakizaki (GK) rats, which were created by repetitive breeding of Wistar rats with the poorest glucose tolerance, develop peripheral insulin resistance without elevated level of plasma fatty acids (Goto et al. 1976). They have thus been used in the investigation of various aspects of T2D not related to obesity. Impaired microvascular function has been reported in GK rats (Padilla et al. 2007), however, in vivo data regarding mitochondrial function in this animal model are still scarce. In a recent study, Macia et al. characterized PCr recovery following a 6‐min electrical stimulation protocol in the gastrocnemius muscle of GK rats. While no change in mitochondrial oxidative capacity was observed in GK rats, it is not clear whether other aspects of muscle metabolism, for example, tissue oxygenation, also remained the same. Further, energy metabolism during the progression of T2D has not been characterized.

The purpose of this study was to examine whether the T2D condition and its progression slows PCr recovery kinetics and alters tissue oxygenation. We sought to assess metabolic response of the skeletal muscle to ischemia‐reperfusion in non‐obese GK rats. Interleaved ^31^P MRS and BOLD MRI acquisitions were performed continuously during an ischemia and reperfusion protocol to evaluate mitochondrial oxidative capacity and muscle oxygenation. Progression of the disease was investigated by conducting experiments on both young and adult GK rats and their age‐matched controls.

## Materials and Methods

### Animals

Non‐obese male Goto‐Kakizaki (GK) rats (Charles River Laboratories, the United States) were scanned at about 12 weeks as the young group (*n* = 7). The same rats were scanned at about 18 weeks as the adult group (*n* = 7). Wistar rats (Charles River Laboratories, the United States) were scanned at about 13 weeks as the young control group (*n* = 6), with five of the same rats scanned at about 20 weeks as the adult group. Additional four adult Wistar rats were also scanned at about 20 weeks of age (*n* = 9 in total). The animal protocol was approved by the Institutional Animal Care and Use Committee of the Case Western Reserve University.

### Experimental protocol

Animals were anesthetized with isoflurane (1.5–2.5%) and placed in a cradle in lateral position. Respiration rate and body temperature were monitored during the experiments. The body temperature was maintained at 35.5 ± 0.5°C by blowing hot air into the MRI scanner through a feedback control system. An inflatable cuff was placed at the thigh of the rat. Ischemia was induced by occluding the femoral artery with the inflated cuff. After the MRI scan, the blood glucose concentration was determined using a glucometer with blood samples obtained from tail vein.

### MRI and MRS studies

All experiments were conducted on a 9.4T Bruker Biospec horizontal scanner (Bruker Biospin Inc., Billerica, MA). A Bruker ^1^H volume coil was used for ^1^H image acquisition. An in‐house built, 20‐mm diameter ^31^P saddle coil was placed around the calf muscles to acquire the ^31^P spectra. The leg was secured to the ^31^P coil that was attached to the cradle to avoid potential motion during the experiment. To minimize the coupling between the ^1^H and the ^31^P coils, the orientation of the two coils were adjusted such that their B_1_ fields were perpendicular to each other.

Scout ^1^H images were acquired to determine the position of the lower leg. Automatic, localized shimming was performed on an isotropic voxel of 20 × 20 × 20 mm^3^ that covered the entire lower leg using a PRESS sequence. A proton linewidth of 120–140 Hz was achieved after the shimming. The experimental setup and shimming took about 15–20 min on average.

**Figure 1:**
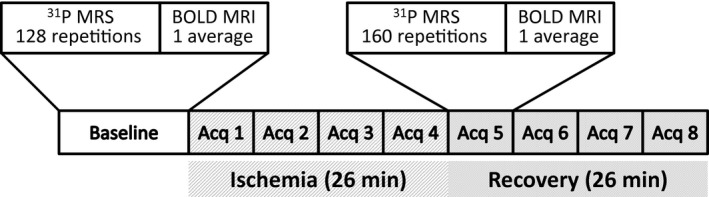
Data acquisition scheme. Interleaved ^31^P MRS and BOLD MRI acquisitions were performed at baseline and during ischemia and reperfusion. The acquisition block was repeated four times each during ischemia and reperfusion, respectively.

One twenty eight ^31^P spectra were acquired, followed by the acquisition of three BOLD images, leading to a total acquisition time of ~7 min at baseline. Ischemia was then induced by inflating the cuff to 300 mmHg pressure within 1–2 sec. After 26 min of ischemia, perfusion was resumed immediately by deflating the cuff. During ischemia and reperfusion, interleaved ^31^P MRS and BOLD MRI acquisitions were performed continuously. ^31^P MRS scan consisted of the acquisition of 160 single‐average ^31^P spectra. Each acquisition used a hard excitation pulse with a flip angle of 60°, followed by the acquisition of the free induction decay (FID) with 600 points and a spectral width of 6 kHz. A 2‐sec interscan delay (TR) was used, leading to a total acquisition time of 5 min 22 sec. BOLD images of an axial slice were acquired using the FLASH sequence. Imaging parameters were: TR, 500 msec; echo time (TE), 7 msec; flip angle, 40°; field‐of‐view, 4 × 4 cm^2^; matrix size, 128 × 128; number of averages, 1. Total acquisition time was 64 sec for each BOLD scan. The interleaved ^31^P and BOLD acquisitions were repeated four times each during ischemia and reperfusion, respectively. The experimental protocol and MR acquisition scheme are illustrated in Figure 1.

### Data analysis

^31^P MRS data were analyzed using in‐house developed software written in MATLAB (Mathworks, Natick, MA). Four ^31^P spectra were averaged to achieve an adequate signal‐to‐noise ratio (SNR) of ~7 for PCr peak at baseline. The averaged spectra were zero‐padded to 2048 points and a 30 Hz line‐broadening was applied before Fourier transform. The transformed spectra were phase‐corrected manually using zero‐ and first‐order correction. The area under PCr was calculated by integration.

Baseline PCr level was calculated as the peak area from the averaged spectra acquired at baseline. PCr level during the entire ischemia‐reperfusion period was normalized to the baseline value. Upon cuff inflation, the initial slope of %PCr depletion was quantified as an index of resting metabolic rate. During reperfusion, the time constant of PCr recovery was estimated by fitting a monoexponential function to the PCr recovery curve (Giannesini et al. 2010). Although the use of a 2‐sec TR introduced signal saturation, the kinetics of the normalized PCr signal was not affected. In addition to the time constant, the initial rate of %PCr recovery was also quantified by calculating the initial slope of %PCr recovery.

P_i_ levels were quantified by averaging 128 spectra acquired at the baseline and at the end of reperfusion, respectively. pH values at baseline and at the end of ischemia and reperfusion were calculated from the chemical shift of P_i_ relative to PCr ($\delta_{P_{i}}$) according to the following equation (Soussi et al. 1990):

$$\text{pH}=6.75+\text{log}\frac{\delta_{P_{i}}-3.27}{5.69-\delta_{P_{i}}}$$

BOLD images were also processed using MATLAB‐based software (Mathworks, Natick, MA). Regions of interest (ROI) that encompassed the tibialis anterior and the gastrocnemius muscles were segmented manually. Signal intensity in these two ROIs, as well as in the entire calf muscle, was calculated as the mean value of all pixels within the ROIs. The signal was then normalized to the corresponding baseline values.

### Statistics

Results are reported as mean ± standard deviation. Two‐way analysis of variance (ANOVA) was used for data comparison. If statistical differences were detected, multiple pairwise comparisons were performed using two‐tailed student's *t*‐test. Significant difference was accepted at *P* < 0.05.

## Results

### Animal characteristics

The age, body weight, and blood glucose level for young and adult groups were summarized in Table 1. The body weight for young and adult GK rats was significantly lower than their age‐matched controls. Blood glucose level was significantly higher than that of the age‐matched controls. Young and adult GK rats were scanned at 12.2 ± 0.3 weeks and 18.1 ± 0.2 weeks, respectively. The age at which the young and adult Wistar rats were scanned was 13.8 ± 0.1 weeks and 20.4 ± 1.4 weeks, respectively.

**Table 1:** Animal characteristics

	Young		Adult	
	Control	GK	Control	GK
Age (weeks)	13.8 ± 0.1	12.2 ± 0.3	20.4 ± 1.4	18.1 ± 0.2
Body weight (g)	389.1 ± 16.2	293.4 ± 37.4^a^	507.5 ± 32.7	356.5 ± 19.9^a^
Blood glucose level (mg/mL)	123.8 ± 6.1	227 ± 7.5^a^	108 ± 23.2	204 ± 6^a^

a *P* < 0.05, GK versus control.

### ^31^P MRS

**Figure 2:**
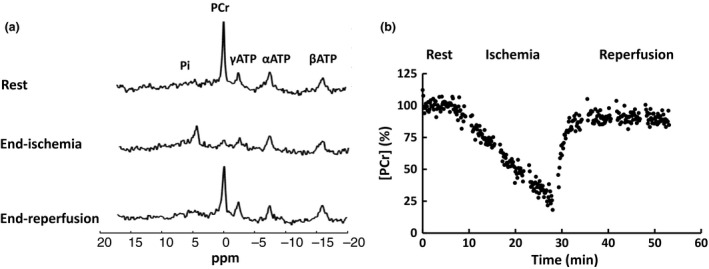
Representative ^31^P spectra at baseline, end of ischemia, and end of reperfusion (a) and changes in PCr concentration (b) during ischemia‐reperfusion with a temporal resolution of 8 sec.

Representative ^31^P spectra at baseline, end of ischemia, and end of reperfusion from an adult control rat are shown in Figure 2A. Each spectrum is an average of four acquisitions, giving rise to a temporal resolution of 8 sec. PCr depletion and P_i_ accumulation were evident during ischemia. A representative time course of PCr changes during the entire experimental protocol is shown in Figure 2B. PCr showed progressive decrease during the entire period of ischemia. There was no difference in the initial rate of %PCr depletion among the four experimental groups (2.9 ± 0.2%/min vs. 2.7 ± 0.4%/min for young and adult controls; 2.8 ± 0.2%/min vs. 2.7 ± 0.2%/min for young and adult GK rats). Upon reperfusion, PCr was rapidly resynthesized, accompanied by the decrease of P_i_ to near‐baseline level.

**Figure 3:**
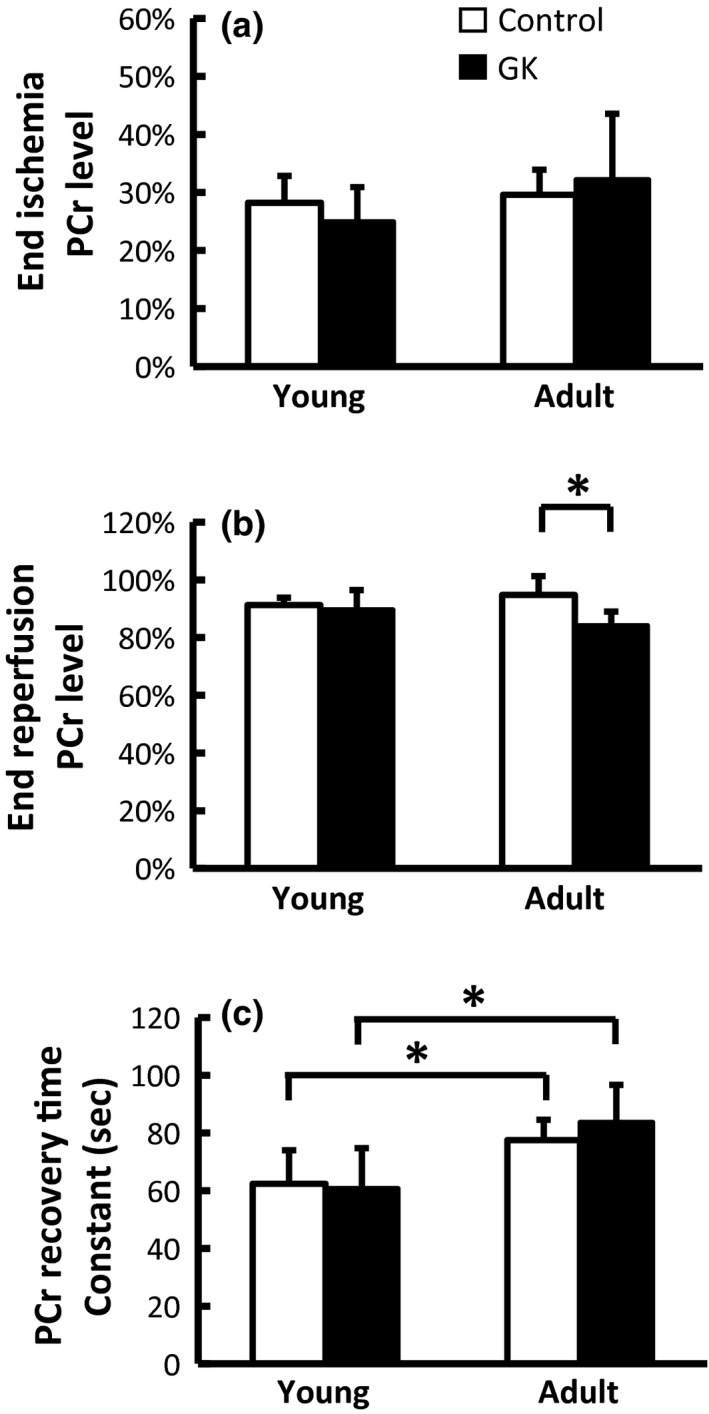
Metabolic response to ischemia and reperfusion measured by ^31^P MRS. (a) Normalized PCr level at the end of ischemia; (b) Normalized PCr level at the end of reperfusion; (c) Time constant of PCr recovery during reperfusion.

PCr levels at the end of ischemia and reperfusion are shown in Figure 3A and B, respectively. All four groups of rats showed significant but similar levels of PCr depletion at the end of ischemia (Figure 3A). PCr levels at the end of reperfusion ranged from 86% to 95%, with the adult GK rats showed significantly lower PCr recovery compared to their age‐matched controls (Figure 3B). No statistically significant interaction between the age and animal strain was detected by ANOVA. Consistent with the incomplete PCr recovery, there was an increase in P_i_ at the end of reperfusion. Again, adult GK rats showed the highest increase in P_i_ compared to their age‐matched controls (8.5% vs. 4.6%). P_i_ increase in young GK and control rats was 4.3% and 5.1%, respectively.

Figure 3C shows the time constant of PCr recovery at the onset of reperfusion. There was no difference between the GK rats and their age‐matched controls. However, the adult rats showed prolonged PCr recovery kinetics for both GK and control rats (*P* < 0.05). There was no statistically significant interaction between the age and animal strain. Consistent with increased time constant of PCr recovery, both adult and GK rats also showed a decrease in the initial rate of %PCr recovery: 1.2 ± 0.2%/sec versus 0.9 ± 0.1%/sec for young and adult controls, and 1.3 ± 0.3%/sec versus 0.9 ± 0.4%/sec for young and adult GK rats, respectively (*P* < 0.05).

**Figure 4:**
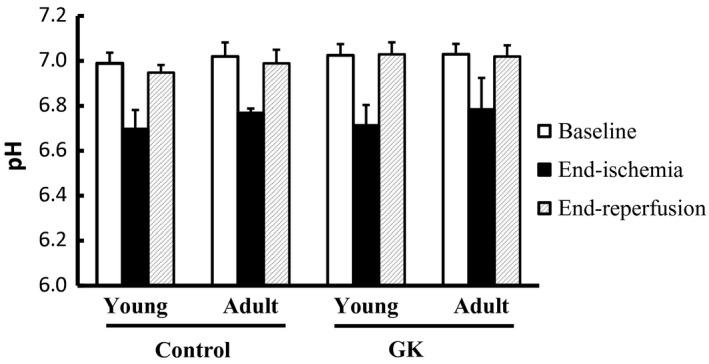
Intracellular pH at baseline, end of ischemia, and end of reperfusion.

As expected, there was a significant reduction in pH during ischemia, and it returned to the baseline level after reperfusion (Fig. 4). No significant difference in pH was found among all four groups.

### BOLD MRI

**Figure 5:**
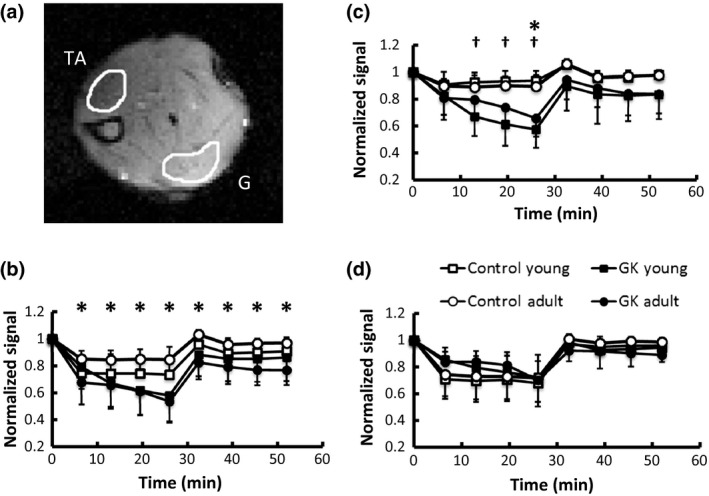
Tissue oxygenation measured by BOLD MRI. (a) A representative BOLD image acquired at baseline with ROIs of tibialis anterior (TA) and gastrocnemius (G) muscles; (b–d) Normalized BOLD signal changes during ischemia and reperfusion in all muscles (b), gastrocnemius (c), and tibialis anterior (d). *P < 0.05, adult GK rats versus adult controls; ^†^ *P* < 0.05, young GK rats versus young controls.

Figure 5 shows baseline‐normalized BOLD signal intensity during ischemia and reperfusion. Ischemia induced a significant decrease in BOLD signal intensity, suggesting decreased tissue oxygenation. Comparing to their age‐matched controls, adult GK rats showed significantly lower BOLD signal intensities during ischemia‐reperfusion, suggesting more pronounced reduction in tissue oxygen level in the entire calf muscle (Fig. 5B). The difference in BOLD signal between the GK rats and the controls was predominantly caused by the difference in gastrocnemius muscle (Fig. 5C). In contrast, BOLD signal in tibialis anterior muscle was similar among all groups during ischemia and reperfusion (Fig. 5D).

## Discussion

In this study, we characterized *in vivo* metabolic and physiological alterations in response to ischemia and reperfusion in the skeletal muscle of GK rats, a rat model of non‐obese T2D. GK rats were shown as one of the best animal models of non‐obese T2D (Akash et al. 2013). In this study, both young and adult GK rats showed significantly higher blood glucose level compared to their age‐matched controls, suggesting that insulin resistance was present at an early age. The time constant of PCr recovery, an index of mitochondrial oxidative capacity, was not significantly different between GK rats and their age‐matched controls. However, we observed a significant reduction in PCr recovery level after ischemia in adult GK rats. Interestingly, ischemia induced a more pronounced reduction in BOLD signal in GK rats, suggesting reduced muscle oxygenation in gastrocnemius muscle during ischemia and reperfusion.

It has been shown that skeletal muscle oxidative capacity can be quantified from the kinetics of PCr recovery following a period of PCr depletion (Lanza et al. 2011). In a recent study using a 6‐min stimulated maximal isometric contraction protocol (Macia et al. 2015), Macia and colleagues observed that the initial rate of PCr resynthesis after the stimulation was not different between the GK rats and the controls. In our current study, PCr depletion was induced by a 26‐min ischemia protocol. The ischemia protocol induced a similar level of PCr depletion comparing to the stimulation study by Macia et al. Further, the time constant of PCr recovery during reperfusion was also similar between the GK rats and the controls in both age groups, suggesting unaltered mitochondrial oxidative capacity. Given that GK rats in both age groups were insulin‐resistant but showed similar mitochondrial oxidative capacity as the controls, it can be expected that pre‐diabetic GK rats will also show normal oxidative capacity. These results confirm the previous observation that mitochondrial impairment is not the causative factor for the development of insulin resistance in GK rats.

Oxygen deficiency during ischemia also induced a significant decrease in intracellular pH. Previous studies have shown that the time constant of PCr recovery after its depletion is inversely related to intracellular pH (de Graaf et al. 2007; Forbes et al. 2009; Layec et al. 2013). Possible mechanisms include direct impact of acidosis on mitochondrial respiration (Harkema and Meyer 1997), ATP consumption during pH restoration (Roussel et al. 2000), and the modulation of creatine kinase equilibrium (Arnold et al. 1984; Walter et al. 1999; Iotti et al. 2005). In their recent study on GK rats (Macia et al. 2015), Macia and colleagues reported an intracellular pH of 6.3–6.4 at the end of 6‐min maximal isometric contraction. The subsequent PCr recovery showed a rate constant of 0.38–0.39 per min, corresponding to a time constant of 154–158 sec. In our current study, ischemia‐induced pH decrease was much less, ranging from 6.7 to 6.8. As a result, the time constant of PCr recovery was much shorter, from 60 to 80 sec. While these results support the negative correlation between the time constant of PCr recovery and intracellular acidosis, it is important to note that the measured PCr recovery kinetics still deviated from that measured under physiological pH, and thus was impacted by both mitochondrial oxidative capacity and proton clearance. Future studies can use a shorter duration of ischemic period to minimize the effect of acidosis.

Previous studies on lean diabetic patients using ^31^P MRS have documented decreased ATP synthesis at resting state and during insulin stimulation (Befroy et al. 2007). Reduction in the number and size of muscle mitochondria has also been documented in obese T2D patients (Ritov et al. 2005), as well as the lean insulin‐resistant offspring of T2D patients (Morino et al. 2005). These observations have led to the hypothesis that mitochondrial deficiency may be responsible for insulin resistance (Lowell and Shulman 2005; Morino et al. 2006). However, normal PCr recovery after exercise/stimulation was also observed in both T2D patients and animal models of obese T2D (De Feyter et al. 2008a,b). Hence, the causal association between mitochondrial dysfunction and the development of insulin resistance remains to be elucidated. The normal mitochondrial oxidative capacity in GK rats supports the notion that insulin resistance is not necessarily associated with impaired mitochondrial oxidative capacity in T2D. On the other hand, we observed a significant reduction in post‐ischemia PCr level in adult GK rats. Consistent with PCr reduction, GK rats also showed more pronounced increase in P_i_ at the end of reperfusion. Previously, incomplete PCr recovery has been observed in normal Wistar rats after a long period of ischemia (6 h) (Morikawa et al. 1991, 1993). However, the duration of ischemia in this study was much shorter. While one cannot rule out the possibility of metabolic dysfunction, the observed reduction in end‐reperfusion PCr level in adult GK rats may also be caused by a deficit in oxygen delivery, as vascular impairment and lower microvascular oxygen pressure have been observed in adult GK rats (Padilla et al. 2006, 2007).

Comparing to the control rats, both young and adult GK rats also showed a more pronounced decrease in the normalized BOLD signal during ischemia. In general, BOLD signal depends on both blood oxygenation and blood volume (Ogawa et al. 1990). During ischemia induced by cuff‐inflation, change in blood volume was minimal. Hence, the observed changes in BOLD signal are more likely associated with altered muscle oxygenation. The differences in baseline normalized BOLD signal could be caused by either a difference at the baseline (the denominator), or by a difference during ischemia (the numerator). A previous study has reported lower baseline microvascular oxygen pressure in GK rats (Padilla et al. 2007), possibly caused by vascular impairment (Padilla et al. 2006). This reduction in baseline muscle oxygenation in GK rats might have given rise to more pronounced reduction in normalized BOLD signal during ischemia observed in this study. Alternatively, this reduction may also reflect increased oxygen extraction by GK rats during ischemia. Impaired glucose uptake in T2D can lead to increased fatty acid utilization for ATP generation, which consumes more oxygen to fuel the oxidation. Indeed, increased fat oxidation has been reported in T2D patients and obese individuals with insulin‐resistant (Felber et al. 1987). Hence, the more pronounced BOLD signal reduction in GK rats during ischemia can also be associated with increased fat oxidation. Which is the dominant mechanism that leads to the observed reduction in normalized BOLD signal in GK rats needs further investigation. Furthermore, the time course of BOLD signal recovery immediately after the onset of reperfusion can also provide important insight into the relationship between tissue oxygenation and muscle energetics.

With measurements of metabolic response from different muscles, BOLD MRI also provided assessment of heterogeneous alterations in muscle metabolism. Interestingly, gastrocnemius muscle in GK rats showed more pronounced BOLD signal reduction during ischemia‐reperfusion compared to the controls. In contrast, oxygen level in tibialis anterior muscle was similar between GK rats and the controls. Comparing to the tibialis anterior muscle, gastrocnemius muscle is predominantly glycolytic type IIb fibers in rats (Ariano et al. 1973; Armstrong and Phelps 1984; Staron et al. 1999). It was reported that glycolytic fibers are more insulin resistant in both rats (James et al. 1985) and humans (Albers et al. 2015). It is possible that gastrocnemius muscle with a larger fraction of glycolytic type IIb fibers is more likely to manifest metabolic alterations than the tibialis anterior muscle in T2D GK rats.

A limitation of this study is that the ^31^P MRS signals were acquired in a nonlocalized fashion and absolute concentrations of phosphate metabolites were not quantified. Hence, they represented the weighted averages from different muscle fiber types and cannot capture the heterogeneous alterations in mitochondrial function. Distinct differences in fiber‐type composition exist among limb muscles, which give rise to metabolic diversity in response to stress and pathophysiological changes, as well as marked disparities in the recovery rate of PCr following its depletion (Lillioja et al. 1987). Recently, spectrally selective PCr imaging has been proved feasible in measuring post‐exercise PCr resynthesis rate in the calf muscles of human subjects with adequate spatial and temporal resolution at 7T (Parasoglou et al. 2012). While ^31^P imaging of rodents is still challenged by the requirement of much higher spatial resolution, combining a spectrally selective PCr imaging method with other fast imaging approaches may enable the monitoring of the kinetics of PCr recovery in different muscle types in vivo.

In conclusion, we have observed unaltered mitochondrial oxidative capacity and decreased muscle oxygenation during ischemia‐reperfusion in non‐obese T2D GK rats. These findings provide evidence that insulin resistance is not accompanied by impaired mitochondrial oxidative capacity in this animal model. The lower BOLD signal during ischemia‐reperfusion may be a consequence of impaired microvascular function and/or substrate alteration resulting in higher oxygen extraction. These findings provide the evidence that insulin resistance leads to altered oxygen utilization without impaired mitochondrial oxidative capacity in GK rats.

## Conflict of Interest

None declared.
